# Retraction: *Arnica montana* Stimulates Extracellular Matrix Gene Expression in a Macrophage Cell Line Differentiated to Wound-Healing Phenotype

**DOI:** 10.1371/journal.pone.0219007

**Published:** 2019-06-20

**Authors:** The PLOS ONE Editors

Following publication of this article [1], concerns were raised about the concentration of *Arnica montana* (*Arnica m*.) used in the experiments, and that the reported gene expression changes are within the range of what would be expected for standard noise within an RNA-seq dataset.

Specifically, the authors reported results of RNA-seq experiments and follow-up cell culture-based studies to examine effects of *Arnica m*. on macrophages, using dilutions of *Arnica m*. ranging from 2c to 15c (i.e. 10^−4^ to 10^−30^). The authors reported that sesquiterpene lactones were present in 2c experiments at a final concentration of 1.05 x 10^−8^; the exact concentration of other components was not reported. In Figs 1 and 2, the article reports an absorption spectrum and nanoparticle spectrum analysis for the *Arnica m*. 1c starting material, but not for the 2c or other solutions used in the study, or for a control solution. This raises concerns on whether there is sufficient evidence to demonstrate that biochemically active ingredients remain in the diluted solutions used in the experiments.

The RNA-seq results obtained using the most concentrated *Arnica m*. solution (2c) are reported in Table 1, wherein mean Log_2_ Fold Change values are reported in the range from -0.36 to 0.3, i.e. the authors reported changes in gene expression in the range of 0.75–1.25-fold following *Arnica m*. treatment as compared to cells in the control group. A number of concerns have been raised about these results:

- Questions have been raised as to whether RNA-seq data within this fold range reflect biologically significant or reproducible changes in gene expression.
- The statistical strength of the results reported in Table 1 and Fig 5 have been called into question, and specifically concerns have been raised as to whether the p values reported in Table 1 provide a valid statistical assessment or clear representation of the relationship between the gene expression mean and standard error values in columns 4–7.
- Follow-up experiments using pooled samples of cells treated with more dilute solutions (3c, 5c, 9c, 15c) yielded results in approximately the same range of fold change, as reported in Fig 5, calling into question the specificity of the reported results.

The *PLOS ONE* Editors have discussed the study design and results reported in this article with experts in RNA-seq analysis, statistical analysis and members of our Editorial Board. Based on our assessment and the advice received, and in light of the above concerns, we have determined that the results presented in this article do not provide sufficient support for claims about effects of *Arnica m*. on gene expression. Hence, we are retracting this article due to concerns about the study design and about the validity and reliability of the reported conclusions. We regret that these issues were not fully addressed during the article’s pre-publication peer review.

In addition to the above, the *PLOS ONE* Editors hereby notify readers that the Competing Interests statement was incorrect for this article and should have explicitly stated that Boiron Laboratories, a company that provided funding support for this study, markets homeopathic products including various dilutions of *Arnica m*.

MM, CB, DO, EG, and PB did not agree with retraction. AB, LB, and FDL did not respond.
